# Electrokinetic Microfluidics at the Convergence Frontier: From Charge-Driven Transport to Intelligent Chemical Systems

**DOI:** 10.3390/mi17010071

**Published:** 2025-12-31

**Authors:** Cheng-Xue Yu, Chih-Chang Chang, Kuan-Hsun Huang, Lung-Ming Fu

**Affiliations:** Department of Engineering Science, National Cheng Kung University, Tainan 701, Taiwan; n98121509@gs.ncku.edu.tw (C.-X.Y.); ccchang0930@gs.ncku.edu.tw (C.-C.C.); n96104496@gs.ncku.edu.tw (K.-H.H.)

**Keywords:** electrokinetic, microfluidic, electroosmotic flow, electrophoresis, dielectrophoresis

## Abstract

Electrokinetics has established itself as a central pillar in microfluidic research, offering a powerful, non-mechanical means to manipulate fluids and analytes. Mechanisms such as electroosmotic flow (EOF), electrophoresis (EP), and dielectrophoresis (DEP) re-main central to the field, once more layers of complexity emerge heterogeneous interfaces, viscoelastic liquids, or anisotropic droplets are introduced. Five research directions have become prominent. Field-driven manipulation of droplets and emulsions—most strikingly Janus droplets—demonstrates how asymmetric interfacial structures generate unconventional transport modes. Electrokinetic injection techniques follow as a second focus, because sharply defined sample plugs are essential for high-resolution separations and for maintaining analytical accuracy. Control of EOF is then framed as an integrated design challenge that involves tuning surface chemistry, engineering zeta potential, implementing nanoscale patterning, and navigating non-Newtonian flow behavior. Next, electrokinetic instabilities and electrically driven micromixing are examined through the lens of vortex-mediated perturbations that break diffusion limits in low-Reynolds-number flows. Finally, electrokinetic enrichment strategies—ranging from ion concentration polarization focusing to stacking-based preconcentration—demonstrate how trace analytes can be selectively accumulated to achieve detection sensitivity. Ultimately, electrokinetics is converging towards sophisticated integrated platforms and hybrid powering schemes, promising to expand microfluidic capabilities into previously inaccessible domains for analytical chemistry and diagnostics.

## 1. Introduction

Microfluidics did not rise overnight; its influence accumulated gradually as re-searchers learned how to confine liquids inside channels narrower than a strand of hair [1]. Once fluids could be guided reliably in these microscale environments, a new style of experimentation began to take shape. Reagents lasted longer because only tiny amounts were needed. Analytical steps that once took hours could suddenly finish far earlier. Often, entire workflows that previously spanned a benchtop could be condensed into a compact lab-on-a-chip arrangement [2,3]. The influence of these platforms has extended well beyond analytical chemistry, reaching applications in materials engineering and biomedical assays [4]. Among available actuation strategies, electrokinetic driving plays a particularly prominent role. Whereas pressure-driven pumping typically yields a parabolic velocity distribution, electric-field–induced transport more closely approaches plug-like flow, a feature that is especially advantageous for preserving spatial resolution and separation performance [5].

Electrokinetic phenomena arise from the interaction between an applied electric field and excess charge residing within the electrical double layer (EDL) at liquid–solid interfaces [6,7,8]. When microchannel materials such as glass or elastomers (e.g., polydimethylsiloxane) are exposed to aqueous electrolytes, surface ionization produces fixed charges that recruit counter-ions from the bulk solution. These charges organize into a compact Stern layer and an adjacent diffuse layer, together constituting the EDL. The electric potential defined at their boundary—the zeta potential (ζ)—plays a central role in determining both the strength and direction of electrokinetic transport [6,7,8].

Electrokinetic motion in microchannels is commonly interpreted through three interacting transport processes rather than strictly separate regimes. Electroosmotic flow (EOF) originates from the action of an axial electric field on counter-ions within the diffuse layer of the electrical double layer, with viscous coupling transferring this motion to the surrounding liquid [9]. Under identical field conditions, charged species may migrate relative to the fluid via electrophoresis (EP), a mechanism central to capillary electrophoresis and related high-resolution separations [10,11]. Dielectrophoresis (DEP) arises under spatially nonuniform electric fields, where field gradients induce forces even on electrically neutral particles. In this case, even neutral but polarizable particles experience a net force, allowing their selective trapping or directional steering based on differences in dielectric response rather than net charge alone [12,13]. Although these three modes originate from a common electrostatic forcing landscape, their mathematical scaling diverges in ways that fundamentally reshape how microfluidic behavior should be interpreted. Across microfluidic platforms, EOF, DEP, electrohydrodynamic instability, and electrokinetic enrichment are often discussed as separate mechanisms. However, these phenomena originate from a shared physical foundation: the interaction between electric fields, spatially distributed charges within the EDL, and fluid–ion coupling under confinement [14]. Variations in field uniformity, interfacial charge regulation, and transport timescales determine whether the response manifests as steady plug-like flow, differential particle migration, nonlinear vortical instability, or localized analyte accumulation.

Electric fields in electrokinetic microfluidic systems actuate both droplets and micro/nano-scale samples, but the underlying constraints fundamentally differ [4]. Droplet motion is dominated by multiphase electrohydrodynamics, where interfacial charge redistribution, deformation, and liquid–liquid coupling introduce instability and control complexity [2]. By contrast, transport of micro/nano-samples proceeds within a single phase and is increasingly constrained by electrical double-layer interactions, Brownian motion, Joule heating, and field-induced dispersion as dimensions shrink toward the nanoscale [10]. It additionally distinguishes electrokinetic behavior in static suspensions—dominated by inherent electrical double-layer polarization and particle–particle interactions—from that in flowing systems, where convection, shear, and field–flow coupling fundamentally modify the resulting forces and transport pathways [15].

EOF [16,17,18,19] and EP [20,21,22,23] both follow a linear electrokinetic regime in which transport velocity scales proportionally with the applied electric field. The motion is primarily dictated by the zeta potential at either the channel wall or the particle surface, linking surface charge conditions directly to the efficiency of field-driven migration. By contrast, DEP [24,25,26,27,28] does not depend on net charge transport; instead, the induced dipole polarization interacts with the field gradient, producing a nonlinear force that scales with the third power of particle radius and with the spatial gradient of the electric field intensity. As a result, the practical dominance of each mechanism is not dictated by field strength alone but by how the geometry, polarizability contrast, and boundary conditions amplify or suppress their respective scaling responses. Table 1 illustrates the mathematical models and mechanisms of these three electrokinetic microfluidic states.

Recent advances continue to refine understanding of these mechanisms inside both microchannels and nanochannels. When characteristic channel dimensions approach the thickness of the EDL, unconventional transport modes can arise and expand the design space for sensing, separation, and energy conversion. The remainder of this review summarizes current progress in electrokinetic microfluidics, beginning with fundamental physical principles and extending toward fabrication strategies for microscale and nanoscale conduits, along with experimental methods used to probe electrokinetic behavior [4].

**Figure 1:**
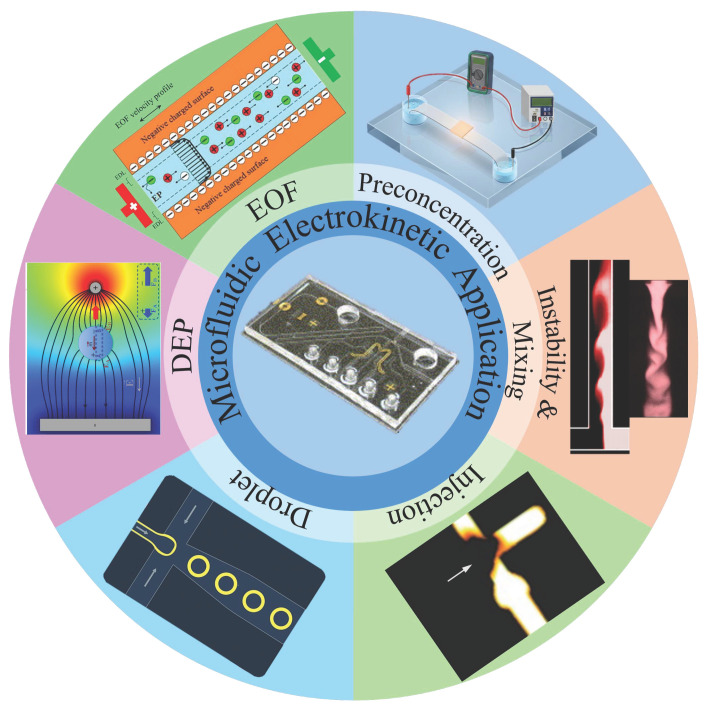
Electrokinetic phenomena and transport in microfluidic systems.

This review is organized around five research directions (Figure 1) that have become particularly active recently. The discussion begins with electrokinetic strategies for manipulating droplets, since droplet control now underpins many digital and compartmentalized microfluidic formats. Attention then shifts to electrokinetic injection, where small differences in how samples enter the system often determine the ultimate separation fidelity. Control of electroosmotic flow is examined next—not only as a fundamental transport mechanism but also as a design variable that reflects how surfaces, interfaces, and chemistry dictate device behavior. Electrokinetic instabilities and their unexpectedly rich mixing dynamics follow as a fourth component. The final section surveys electrokinetic preconcentration approaches that amplify weak signals and enable trace-level detection. Accordingly, this review organizes recent advances not as isolated techniques, but as interconnected electrokinetic regimes that evolve from linear transport to nonlinear instability and field-amplified enrichment as geometric confinement, charge heterogeneity, and electric field gradients intensify. These research directions show that nonlinear electrokinetic behavior is no longer limited to describing how charge moves in confined liquids. Gradually, it has emerged as a valuable engineering tool for sensing, purification, and the microscale management of cells, microorganisms, and biomolecules [29,30,31,32,33]. The field is still far from saturated; several technical barriers continue to slow translation into broader device platforms. Even so, the pace of recent progress suggests that electrokinetic strategies will continue to unlock new analytical capabilities and influence the way microscale systems are designed in the coming years.

## 2. Electrokinetic Control of Droplets

**Figure 2:**
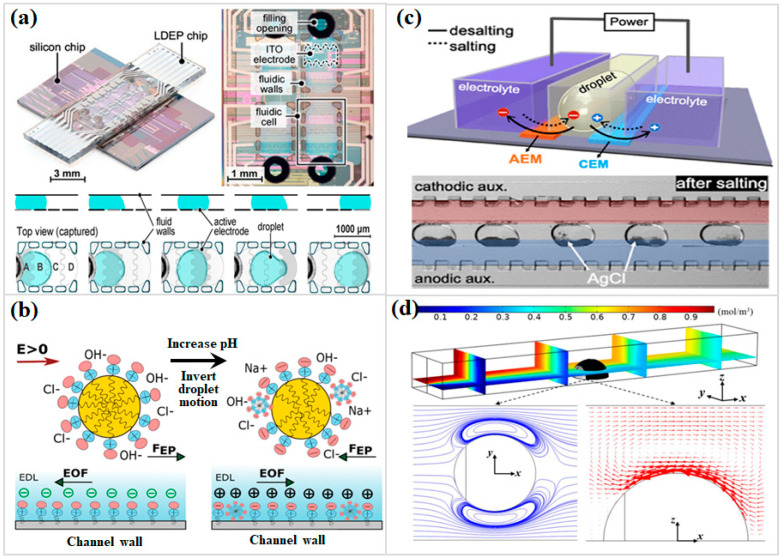
(**a**) Liquid DEP microchip and droplet transport phenomena (Reprinted from Ref. [35]). (**b**) Schematic illustration of pH-Tunable electrokinetic movement on the EP and EOF of droplets. (Reprinted from Ref. [38] with permission of Royal Society of Chemistry). (**c**) Schematic illustration of the operating principle of electrokinetic desalting and salting of droplets and photos of in-droplet AgCl precipitation (Reprinted from Ref. [37]) with permission of American Chemical Society. (**d**) Numerical simulation of concentration and flow field distribution on the Janus droplet surface. (Reprinted from Ref. [55]).

In many microfluidic studies, droplets suspended in an immiscible carrier phase are used instead of continuous streams, because reactions and sample chemistry tend to remain more stable when handled in isolated liquid packets [34]. This droplet-based format, commonly referred to as digital microfluidics (Figure 2a), maintains the identity of the sample without the dilution and uncontrolled spreading that frequently accompany bulk flow [35,36]. Cross-contamination is also far less likely, because each droplet behaves as an independent reaction domain that functions as a confined microreactor where reactions proceed in isolation and remain physically well-defined [37]. Within such systems, electrokinetic forces have shown particular value. Because the driving mechanism is electrical rather than mechanical, a broad range of droplet operations—directed transport, coalescence, division, and internal mixing—can be executed with relatively simple hardware and excellent controllability [38,39].

### 2.1. Fundamental Droplet Motion and Control

The electrokinetic velocity of an individual droplet arises from the combined contributions of the EP of the droplet itself and the EOF of the surrounding medium [40,41,42]. The net effect depends strongly on interfacial charge. Surfactants are an effective means to tune this charge. Amphoteric surfactants, for example, possess both cationic and anionic head groups, enabling their net charge to shift dramatically with pH. When the surrounding environment shifts from acidic toward alkaline pH, the sign of the droplet surface charge may also flip, and this reversal can send the droplet migrating in the opposite direction once an electric field is applied. The balance between EOF and EP is usually dynamic in real microchannel environments. The two effects can push a droplet in different directions, and whichever dominates at a given moment sets both the sign and magnitude of motion. Close to the isoelectric point of a surfactant, even modest shifts in concentration can alter this balance enough to redirect droplet travel. Larger droplets also tend to move faster, a trend often linked to stronger local ζ-potential development and wall-mediated strengthening of interfacial forces. These features make pH-dependent separation a realistic and sometimes compelling design route in systems that naturally host pH gradients, including those found in biological fluids (Figure 2b) [38]. Another practical detail cannot be ignored: additives such as Tween 20 can dampen electroosmotic and electrophoretic mobilities to a surprising degree, and this effect frequently becomes a decisive factor when selecting materials and operating conditions for microfluidic assays [43,44,45].

Beyond simple translation, electrokinetic behavior at the droplet interface is equally critical. Simulations illustrate that the velocity fields within both the non-conductive carrier phase and the aqueous droplet can be independently modulated by adjusting ζ-potentials at different interfaces [39]. The resulting direction of movement reflects the balance between ζ-potential at the two-liquid boundary and the microchannel wall. Phase height differences also reshape shear distribution and significantly alter the droplet’s overall velocity [46,47], whereas internal droplet viscosity exerts only minor influence [39,48].

A persistent bottleneck in droplet microfluidics lies in modifying droplet composition after formation. A recently demonstrated approach integrates two ion-exchange membranes (Figure 2c) to drive in flux either into (salting) or out of (desalting) water-in-oil droplets [37]. Precipitation indicators or fluorescent tracers permit direct visualization of ion movement. The ion transfer rate scales linearly with both the applied field and ionic strength, and desalting efficiencies approaching 98% have been reported. This strategy substantially expands the available design space for droplet-based sample processing and introduces a powerful route for refining droplet content post-generation.

### 2.2. Janus Droplets: Anisotropic Particles with Unique Behaviors

Research on electrokinetic manipulation often returns to Janus droplets [49,50] as a particularly revealing example. The term originates from the dual-faced Roman god, and a detailed examination of the physical structure reveals an unusually precise analogy. In many studies, the droplets are produced by coating only part of an oil droplet with charged nanoparticles, such as aluminum oxide, leaving one hemisphere electropositive while the opposite side becomes electronegative [51,52,53]. This asymmetry does more than create a visual contrast; it establishes a directional electrical identity across the interface, and the resulting anisotropy produces electrokinetic responses that can be adjusted with far greater subtlety than those observed in uniformly charged droplets.

During fabrication, the nanoparticle coverage can be tuned by adjusting the nanoparticle concentration or the applied DC field strength [51]. Electrically induced Janus droplets (EIJDs) reveal a nonlinear dependence of electrophoretic mobility on electric field magnitude because the nanoparticle layer does not maintain a constant packing density. At high field strength, the coverage tends to decrease. When the field varies in time, the lag in coverage relative to field variation produces a velocity hysteresis that has been confirmed experimentally [52]. The velocity-field relationship therefore evolves into a curved, memory-like response rather than a direct proportional scaling.

Extensive modeling and numerical simulation have examined these droplets in detail, integrating force balance, interfacial charge distribution, and double-layer effects. Parametric studies indicate that stronger electric fields, larger zeta potential contrast, higher viscosity ratio between phases, and greater nanoparticle coverage all contribute to faster droplet motion. The droplet-to-channel size ratio behaves differently: velocity first declines as confinement increases, then sharply rises once confinement dominates droplet deformation. These findings match experimental measurements and have enabled systematic mapping of the role of electric field strength, surface coverage, droplet size, and electrolyte formulation [51,53].

More recent work uncovered a new droplet breakup mode when an EJID enters a constricted microchannel under electrokinetic forcing. For droplets with lower permittivity than the continuous phase, the release of dielectrophoretic stress interacts with interfacial energy to trigger breakup within a specific window of constriction ratio and droplet diameter [54]. This duality also reshapes how microfluidic control can be engineered. Rather than viewing charge asymmetry as something to minimize, the anisotropic nature of Janus droplets enables the charge contrast to serve as a functional design variable. In practice, this feature expands the available landscape for droplet manipulation strategies and introduces a new class of electrokinetic configurations that deliberately exploit, rather than avoid, directional electrical imbalance.

### 2.3. Droplets as Tools for Microfluidic Mixing

Janus droplets provide an unusually effective route for microscale mixing, since their asymmetric surface charge naturally introduces counter-rotating electroosmotic flows once a DC field is present (Figure 2d). These opposing motions build persistent vortices along the droplet interface, compressing diffusion length scales and accelerating molecular interpenetration inside narrow channels [55]. Simulation data further indicate that stronger zeta potential contrast and an expanded downstream hemisphere amplify this behavior, strengthening vortex intensity and ultimately improving mixing output. In a complementary direction, contact charge electrophoresis in three-dimensional digital microfluidic configurations drives droplets into controlled horizontal or vertical reciprocation. The resulting oscillation continuously perturbs the internal flow field, and high-speed colorimetric tracking provides direct evidence of rapid internal homogenization over short time windows [41].

Electrokinetic droplet control has also been adapted for oil-in-water emulsions of interest to environmental surveillance following crude oil release. Concentration polarization across nanoporous membranes pre-enriches ionic PAHs in the water compartment, and downstream electrokinetic separation subsequently directs micron-scale oil droplets into discrete outlets [56]. In addition, coupling AC electrothermal excitation with DEP on floating electrodes generates a tunable micropatterning environment capable of routing larger droplets with high spatial accuracy [57]. Asymmetric alternating current electrothermal-induced vortices then disturb the liquid interface aggressively, driving complete mixing within short downstream distances. These combined electrokinetic strategies illustrate how droplet actuation and intensified microscale mixing can function as fundamental operational pillars within next-generation multifunctional lab-on-chip systems.

## 3. Electrokinetic Sample Injections

Electrokinetic injection is widely adopted for sample introduction in microfluidic electrophoresis, where a short voltage pulse drives a defined fraction of analyte from a reservoir into the separation channel [58,59,60]. Although robust, volumetric precision becomes difficult as the plug volume approaches the nanoliter regime. An alternative concept, termed acupuncture injection, was developed for PDMS systems and relies on direct mechanical delivery. A sample-loaded needle punctures the elastomer channel, and a sharp, well-delimited plug (≈3 nL) is introduced simply through controlled syringe pressure. This strategy reduces sample loss, avoids complex voltage-switching circuitry, supports serial multi-sample loading at arbitrary channel positions, and functions regardless of ionic composition during introduction. A single straight channel can function as a dual-mode separation domain, capable of operating capillary zone electrophoresis or electrochromatography simply by adjusting the applied field conditions. The conceptual boundary between the two modes becomes fluid, allowing a single geometry to shift analytical behavior without mechanical reconfiguration. Pursuing even smaller injection plugs, researchers later adapted an “acupuncture injection combined with electrokinetic injection” strategy, where the physical penetration of the channel inlet is paired with electrically driven transfer, producing sharply confined sample introduction at scales difficult to achieve with conventional electrokinetic injection alone. Another study used a combination of needle injection and electrodynamic injection to successfully separate a mixture of fluorescein isothiocyanate and fluorescein via capillary zone electrophoresis (CZE), demonstrating its high-resolution analytical capability [10].

**Figure 3:**
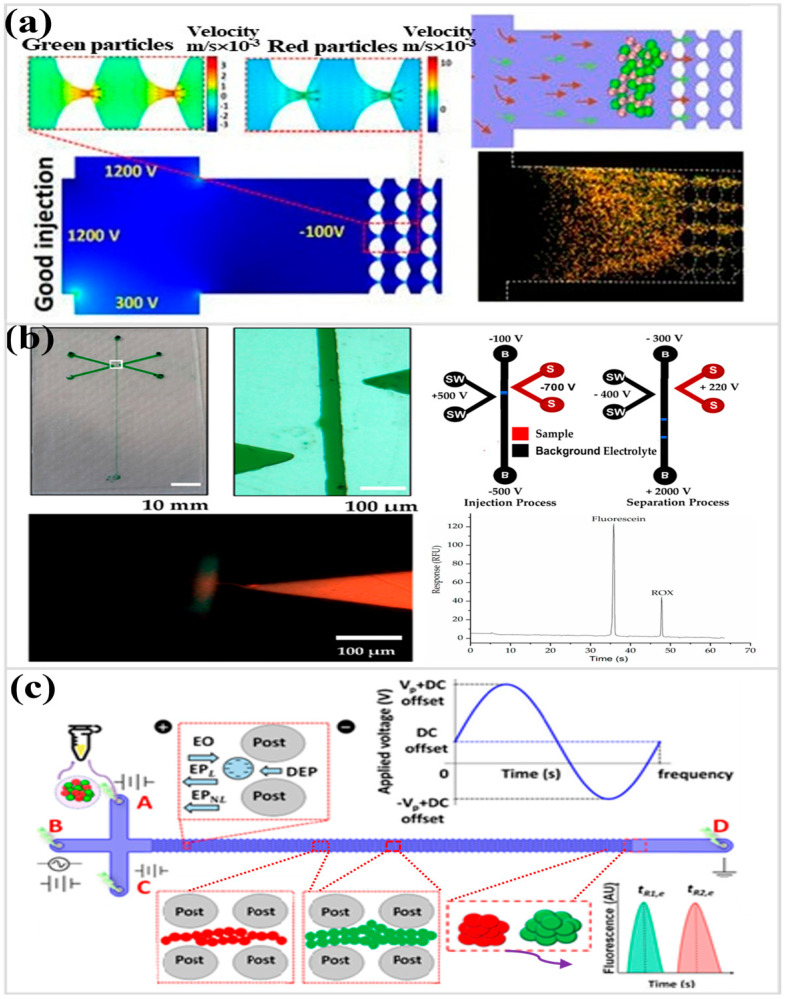
(**a**) A good electrokinetic injection method for the particles entering the post array using numerical simulation and experimental methods (Reprinted from Ref. [58]). (**b**) Photographs of a dual-V hybrid PMMA/tape device, demonstrating the extraction, capture, and detection of small molecules (Reprinted from Ref. [61]). (**c**) Schematic illustration of a DC-biased AC-stimulated microfluidic device for the electrokinetic injection process (Reprinted from Ref. [47]).

Electrokinetic injection exerts a strong influence on final separation quality. The volume introduced, the instant the field is switched, and the physical manner in which the sample thread crosses the channel intersection collectively shape the resolution obtained downstream (Figure 3a). In practice, even modest shifts in electric field strength can redirect particle movement in unexpected ways, occasionally altering transport polarity or producing subtle instability during the gated entry phase [58]. A refined injection process must therefore account for nonlinear electrokinetic effects during the gating phase, ensuring that the sample enters the separation channel cleanly, without distortion, backflow bias, or uncontrolled volumetric spread, ultimately enabling highly discriminative separation performance. A later investigation [59] treated electrokinetic injection not as a fixed operation but as a controllable element that can be tuned on demand. By trimming plug volume, adjusting the spacing between sequential plugs, and altering the moment of channel entry, dispersion was suppressed and separation contrast at the microscale remained high. Under well-paced voltage transitions, nanoliter samples can be transferred with sharply bounded interfaces, while junction leakage and slow backfilling are largely avoided. Comparative evaluation against mechanically assisted injection also revealed where the limits imposed by field-driven forcing begin to dominate, helping define more realistic boundaries for stable, reproducible injection in microfluidic electrophoresis.

Electrokinetic architectures are evolving from traditional sample introduction schemes to field-guided interfaces where sample purification, enrichment, and separation occur simultaneously, representing a key milestone in achieving an automated lab-on-a-chip (LOC) architecture [22,47,61,62,63,64]. For example, in PMMA devices embossed directly into rigid substrates, dielectric-breakdown nanojunctions define a narrow passage where ionic mobility and steric exclusion quietly negotiate who may enter (Figure 3b). Blood cells and large proteins remain behind, while fluorescamine-labeled aminoglycosides advance, accumulate, and finally separate. The offset double-V layout employs a broader gate at 1 µA to admit small pharmaceutical ions, followed by a tighter junction at 0.1 µA that traps them into a compact plug, producing a near thirty-fold gain and therapeutic-range detection for gentamicin, amikacin, and tobramycin [61]. Electrokinetic injection therefore acquires an interpretive role: channel geometry, surface charge, and field asymmetry translate into analytical clarity. A related current-biased strategy reshapes the motion of microbial suspensions, revealing how voltage gradients and hydrodynamic quiet zones divide species with nearly indistinguishable physicochemical profiles (Figure 3c) [47].

In addition, electrokinetic injection provides a direct pathway to introduce trace-level analytes into microscale separation domains, where electrical fields govern volume transfer with minimal moving parts and reduced hardware complexity. Such injection modes allow microscale systems to eliminate conventional mechanical valves and pumps while maintaining highly stable, field-defined mass transfer. The robustness of electrokinetic control becomes especially valuable for low-copy nucleic acids and small fragments in complex biological matrices, enabling concentration, purification, and isolation under transient field conditions [62]. Recent palmtop microchip electrophoresis analyzers further extend this concept toward truly portable chemical analysis, integrating miniaturized optics, HV sources, and compact sample handling units within handheld volumes [63]. Emerging sample-to-answer microfluidic architectures couple extraction, isothermal amplification, and electrophoretic readout into a single platform to accelerate pathogen detection, reduce operator burden, and maintain high analytical specificity near point-of-care environments [64]. The overall direction shows a move toward leaner, electrically controlled analytical systems, with field-driven injection as the most important enabling mechanism.

## 4. EOF and Its Modulation

EOF has long served as a defining mechanism in electrokinetic microfluidics, largely because liquid motion can be generated without pumps or moving structures [65,66,67,68,69]. Once an electric field is imposed, the net charge inside the EDL responds, and the entire fluid column is carried forward [70,71,72,73,74]. The direction and strength of this motion rarely remain constant; they shift with the chemical nature of the channel surface, the ionic composition of the medium, and even small variations in local field intensity. Among these factors, the zeta potential exerts the strongest leverage, acting as the electrochemical reference that links interfacial charge to the velocity profile observed within confined geometries.

### 4.1. The Role of Zeta Potential and Surface Heterogeneity

The zeta potential serves as a key indicator of the electrical nature of the EDL and exerts a decisive influence on electroosmotic transport. Changes in its size or polarity along a microchannel wall can change the flow field, which would otherwise be uniform, into complex and spatially dependent velocity distributions. Such non-uniformity, often introduced by surface patterning, chemical adsorption, or local charge defects, modifies the fluid momentum transfer and reshapes the transport profile compared with a homogeneously charged interface [75,76,77]. Even small differences in surface potential can cause secondary recirculations or localized vortices. This phenomenon shows how sensitive EOF is to interfacial heterogeneity at the microscale. Numerical modeling has revealed that altering the wall zeta potential—whether increasing, decreasing, or following a parabolic gradient—can profoundly reshape the volumetric flow rate within microchannels [78]. Under practical flow conditions, spatial variations in surface potential often develop spontaneously as the moving liquid exchanges ions with the channel walls. These subtle interfacial imbalances contribute to the rise of complex electrohydrodynamic interactions, the behavior of which remains difficult to capture fully within existing theoretical frameworks. Even without an externally applied electric field, a two-dimensional gradient in zeta potential can drive rotational electric body forces near the electrical double layer, giving rise to localized swirling flows and streamwise vortices. This phenomenon points to a plausible electrokinetic origin of vortical structures typically associated with turbulent boundary layers, even under laminar flow regimes. Moreover, uneven charge distributions along the walls may elevate local shear stresses—an aspect of particular concern in shear-sensitive operations such as cellular manipulation, biosensing, and biomedical device design [6,79].

**Figure 4:**
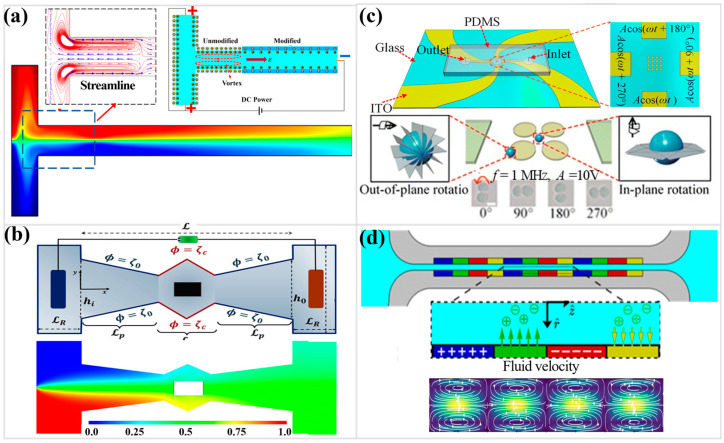
(**a**) Schematic diagram of the non-uniform zeta potential of microchannel structure, the operating concept of micromixing, and the numerical simulation result (Reprinted from Ref. [80]). (**b**) Schematic diagram and numerical simulation result of an electrodynamic mixer with a rhombic geometry microcavity (Reprinted from Ref. [105] with permission of American Chemical Society). (**c**) Schematic illustration of the 3D cell rotation operating principle and photographs of cells rotating in 3D under a microscope (Reprinted from Ref. [114] with permission of Royal Society of Chemistry). (**d**) Schematic illustration of the capillary with periodically patterned active-charged surfaces and numerical simulation result (Reprinted from Ref. [117]).

The deliberate engineering of zeta-potential landscapes thus provides a versatile approach to flow manipulation. For example, electrokinetically generated vortices in a T-junction geometry can be used to make micromixers (Figure 4a), where the surfaces have non-uniform but uniformly charged potentials [80]. Designing the downstream segment with a lower zeta potential creates uneven flow recirculations in a DC field, which greatly improves mixing efficiency. Computational analyses indicate that narrower channels and smaller ratios between modified and unmodified zeta potentials intensify vortex strength, while extending the modified section further improves the mixing performance. These seemingly simple surface modifications translate into sophisticated, self-driven micro-mixers. Three-dimensional simulations [81] further corroborate that charge heterogeneity along the walls can generate robust electrokinetic vortices, substantially accelerating the dispersion and transport of passive species, with the enhancement strongly governed by both surface pattern geometry and the applied field strength.

### 4.2. Influence of Fluid Properties and Channel Geometry

The properties of the working fluid profoundly affect EOF. In many biological and chemical applications, the fluid is not a simple Newtonian electrolyte but may be non-Newtonian [82,83], viscoelastic [84,85,86], a concentration gradient or polarization [87,88,89], magneto-fluidic [90,91,92], or contain additives that alter the interfacial properties [43,93,94]. For example, the commonly used surfactant Tween 20 is often used to reduce particle adhesion, but studies have indicated that it significantly reduces electroosmotic migration in PDMS microchannels. Increasing the Tween 20 concentration continues to reduce mobility, suggesting it should be used with care in applications where flow rate is a critical parameter [43]. Triton X-100, a nonionic surfactant, disrupts hydrophobic interactions at solid–liquid interfaces, thereby limiting particle attachment and reducing surface fouling within microfluidic channels [95]. Sodium dodecyl sulfate (SDS) inhibits particle attachment by imparting strong electrostatic repulsion, but its pronounced protein-disruptive behavior makes it unsuitable for assays requiring preserved biomolecular structure [96].

The rheology of the fluid is also a major factor. Studies on combined pressure-driven and electrokinetically mediated flow of immiscible Newtonian and viscoelastic fluids have shown that the viscoelastic parameter (Weissenberg number) enhances the axial velocity and flow rate. These complex fluid systems also involve intricate thermal transport, where Joule heating and viscous dissipation significantly alter temperature profiles and entropy generation [97,98]. For non-Newtonian fluids described by a power-law model, mixing behavior in channels with periodic zeta potential and sinusoidal wall roughness is complex. The sinusoidally charged surface increases the interfacial contact area, and mixing efficiency can be improved by increasing wall corrugation, Debye length, and the flow behavior index [82,99,100,101]. However, these changes often result in a larger pressure drop. A parametric study is often necessary to identify an optimal design that balances high mixing efficiency with a moderate pressure drop [102,103,104]. Similar trade-offs are observed in nozzle-diffuser-shaped microchambers, where optimizing for mixing efficiency must be balanced against the resulting pressure drop, especially for viscoplastic fluids like Herschel–Bulkley fluids (Figure 4b) [105].

Hydrophobic microchannels with patterned surfaces generally exhibit behavior that defies simple scaling. A modest degree of surface slip can accelerate both the liquid flow and ion transport, producing faster migration than predicted by classical no-slip theory [106,107,108]. When the surface charge becomes uneven, however, the resulting electroosmotic backflow counteracts the driving stream, redistributing momentum and enhancing transverse mixing instead of merely hindering motion. The overall performance reflects a subtle coordination among slip length, Debye screening, channel shape—particularly taper or conical angle—and the magnitude of the zeta potential, each factor responding differently across Newtonian, shear-thinning, and shear-thickening fluids [109]. Studies extending to porous or corrugated channels filled with structured fluids further reveal that microscopic roughness alters flow rate, microrotation, and charge transport in intricate ways, a finding now being exploited for refined microfiltration and selective fluid handling [110].

### 4.3. Advanced Control and Novel EOF Phenomena

Dynamic regulation of EOF has drawn considerable attention beyond conventional surface modification strategies [111,112,113]. A recent design that uses electrokinetic stacking creates a tightly focused enrichment zone (Figure 4c) that amplifies weak cellular responses and reveals subtle dielectric contrasts essential for microfluidic analysis [114]. By integrating induced-charge electroosmosis, travelling-wave DEP, and electrorotation, the platform stably captures individual cells, enhances rotational torque, and enables precise 3D interrogation. In addition, traveling-wave DEP regulates single-particle transport via a frequency-dependent interaction between Coulomb and dielectrophoretic forces. This balance determines directional motion, establishes a size-sensitive transport threshold, and offers a mechanistic foundation for the predictable, selective manipulation of individual particles in electrokinetic micro- and mesoscale systems [115]. The combined forces elevate signal strength by several fold, allowing dielectric shifts, membrane capacitance changes, and cytoplasmic conductivity differences to emerge with unusual clarity. In practice, the gain in signal clarity often proves decisive; subtle electrical or biochemical shifts can suddenly reveal differences that matter in single-cell characterization, drug-response behavior, or other high-throughput biophysical assessments.

The fundamental understanding of EOF continues to evolve. Recent work has developed a hydrodynamic description of self-generated electrolyte flow in capillaries where non-uniform distributions of charge and non-uniform active ionic fluxes coexist [116]. When these two boundary mechanisms are present simultaneously and are spatially offset, they can lead to a symmetry-broken state with steady unidirectional and circulatory flow components, even without any externally imposed fields. For example, these characteristics can be realized by enzyme-coated patches that catalyze reactions to produce local ion fluxes, which then interact with a non-uniform EDL (Figure 4d) to generate bulk fluid motion [117]. This process provides a theoretical framework for understanding self-sustained flows in biological and soft matter systems.

The interaction of EOF with magnetic fields also presents opportunities [118,119]. In such systems, a slip-dependent zeta potential model [120,121] can be more accurate than a slip-independent one. Analytical expressions show that accounting for slip-dependent zeta potential predicts a rise in fluid velocity with increasing slip parameters and Hartmann numbers [122]. A refined electrokinetic description reshapes the predicted thermal response. Once the zeta potential model is adjusted, the temperature field and entropy generation change significantly, with the effect becoming more pronounced when viscous dissipation contributes significantly to heat production. This illustrates the value of accurate physical modeling, as slip-dependent zeta potentials have been shown to significantly improve the electrodynamic energy conversion efficiency in nanochannels compared to slip-independent models [123,124]. Plasma processing can be used to change the surfaces of silicon, which can make the zeta potential higher while keeping the slip length in equilibrium. This process can lead to record streaming potential generation [125]. Light illumination pretreatment can amplify the disparity in zeta potential between viable and non-viable microalgae, allowing cell viability to be distinguished with far greater sensitivity [126]. The underlying mechanism stems from the photosynthetic activity of living cells, whereas dead cells remain electrostatically inert.

## 5. Electrokinetic Instability and Microfluidic Mixing

Building on EOF modulation strategies discussed in Section 4, efficient mixing in confined microchannels remains a persistent experimental difficulty. The narrow dimensions limit fluid motion to very low Reynolds numbers, where viscous stresses become dominant and inertia becomes insignificant. Under these circumstances the streamlines remain ordered, and solutes mix only through molecular diffusion—a process that proceeds at a painfully slow pace. For chemical assays or microscale synthesis, this limitation can severely delay reaction kinetics and reduce analytical throughput [127,128]. To achieve rapid mixing, the flow field must be disturbed so that the interface between adjacent streams continually stretches and folds. One promising route is to exploit electrokinetic instabilities, which use an external electric field to generate vortical motion and fine-scale stirring. Even at vanishing Reynolds numbers, such instabilities can drive vigorous, turbulence-like mixing [129,130,131].

### 5.1. Physics of Electrokinetic Instability

**Figure 5:**
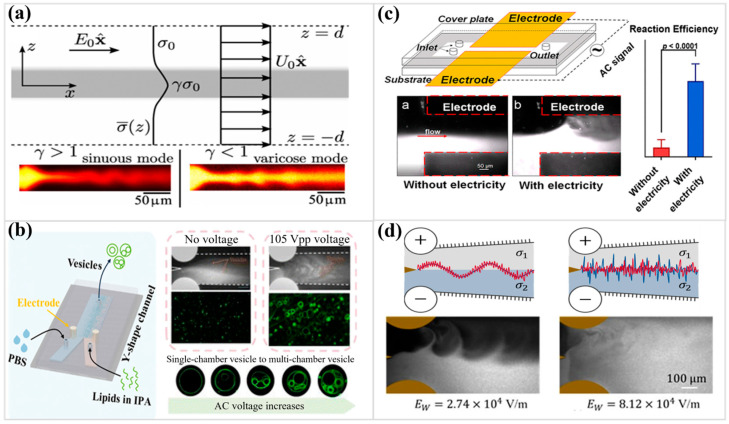
(**a**) Schematic illustration of the base state of electrokinetic instability with coflowing sheath and center streams with different electrical conductivities, and experimental results for sinuous and varicose modes (Reprinted from Ref. [133] with permission of American Physical Society). (**b**) Schematic diagram of the electrokinetic turbulent microreactor and experimental results for the phospholipid vesicle synthesis (Reprinted from Ref. [139] with permission of Royal Society of Chemistry). (**c**) Schematic diagram of a 3D microfluidic electrokinetic turbulent mixer chip and experimental results of mixing effects with/without an electric field (Reprinted from Ref. [128] with permission of American Chemical Society). (**d**) Schematic diagram of the evolution of electrodynamic flow under an enhanced electric field and visualization results of experimental flow (Reprinted from Ref. [135]).

Electrokinetic instability emerges once a strong direct-current field encounters a medium marked by conductivity or permittivity gradients [132,133,134,135]. When two such streams travel side by side in a narrow microchannel, interfacial charge gradually accumulates, forming a thin electrically polarized layer [133]. Once the imposed field grows strong enough, the resulting body force exceeds viscous resistance, and the once-laminar interface begins to distort, marking the onset of instability. Once this mechanical balance is lost, the interface begins to deform, and oscillatory disturbances evolve into a train of vortices and interfacial waves (Figure 5a) [133,134,135,136].

Flow stability is commonly assessed through the electric Rayleigh number—a dimensionless parameter comparing the ratio of electric body forces to viscous and diffusive damping forces [131]. Instability is triggered when this number exceeds a critical value. A refined form of the electric Rayleigh number—derived using a hyperbolic tangent model for the conductivity distribution—has been shown to better align with experimental data, suggesting that electrokinetic flows in divergent microchannels may be considerably more unstable and therefore more efficient for mixing than previously anticipated [137]. The instability’s morphology and temporal growth strongly depend on system configuration. In multi-stream arrangements, the perturbations can manifest as either sinuous or varicose modes: the former dominates when the central stream is more conductive, while the latter prevails when it is less so. Linear stability analyses have revealed that this mode selection originates from stationary convection cells established by the coupling between the electric field and the induced charge distribution [133].

Electrokinetic instability can also arise in microflows where the electrical conductivity varies along the channel axis, such as during sample stacking in electrophoretic separations. In such configurations, the spatially non-uniform electroosmotic velocity field induces secondary recirculating vortices that progressively rotate the axial conductivity gradient until it becomes transverse to the applied field. Once the imposed field surpasses a critical magnitude, this reoriented gradient acts as a seed for interfacial perturbations and ultimately drives the onset of instability. The predicted threshold electric strengths and the associated flow morphologies obtained from numerical simulations exhibit strong agreement with direct microscopic flow visualizations [138]. The nonlinear evolution of this process shares dynamical features with the Lorenz system, indicating that even small conductivity disparities are capable of producing chaotic oscillations through intrinsic nonlinear coupling among electric field, space charge, and fluid motion [132].

### 5.2. Electrokinetic Effects for Mixing

Chaotic vortices arising from electrokinetic instability provide an effective route to achieving intensive microscale mixing, forming the foundation of high-performance active micromixers [129,130,131]. An electrokinetic turbulent micromixer (Figure 5b) has been used for the controllable synthesis of phospholipid vesicles via nanoprecipitation. By applying a transverse electric field over the co-current solvent and antisolvent streams, rapid mixing can be induced, thereby synthesizing vesicles with controllable size and morphology in less than a second [139]. A detailed exploration of a Y-type electrokinetic micromixer [80] operating in the turbulent regime has revealed a finely balanced set of control factors. The device achieves nearly complete homogenization, reaching a mixedness level of about 93% within 100 ms when the electric field strength, AC frequency, conductivity ratio, and channel width are all set to their best values. Intriguingly, the configuration yielding optimal mixing does not coincide with the most unstable state predicted by linear stability theory, indicating that advanced computational turbulence models are essential for interpreting and guiding mixer design at elevated electric Rayleigh numbers [127].

Electrokinetic micromixer [140,141,142,143,144,145] architectures accelerate fluid homogenization, thereby strengthening mass transfer and markedly improving the efficiency and analytical sensitivity of subsequent biochemical reactions. A study using an electrokinetic turbulent micromixer (Figure 5c) for the glucose oxidation reaction showed that the enhanced mass transfer directly improved the enzymatic reaction efficiency [128]. Intensified microscale convection markedly enhanced the analytical response of glucose assays in both solution and whole-blood matrices. This improvement comes from the more even distribution of reactants and the faster exchange of molecules in the detection zone, which makes the electrochemical signals more stable. The observation underscores the decisive influence of local mixing dynamics on sensitivity and reliability, particularly for applications in point-of-care testing and liquid biopsy analyses. Furthermore, alternating-current electrokinetic agitation [146] has been adapted for the microsynthesis of metal–organic frameworks. A confined aqueous environment, when energized by an applied field, fosters crystal nucleation and growth with remarkable control. Dimensions and morphology become adjustable parameters, achieved through a clean, water-driven route that avoids the complexity and environmental burden of conventional synthesis.

Beyond DC field-induced electrokinetic instability, AC electric fields [147,148,149,150,151] offer a rich parameter space for fluid manipulation. The response of electrokinetic flow to AC fields is frequency-dependent. At lower excitation frequencies, the interfacial layer follows both its intrinsic relaxation frequency and the applied alternating field, whereas at higher frequencies, the response remains confined to its natural resonance. The onset of chaotic and turbulent motion may arise through several nonlinear pathways, such as period-doubling cascades or subcritical bifurcations (Figure 5d) [135]. Even when the two liquid phases possess identical conductivities, effective mixing can still be induced under alternating current by employing conductive channel walls. In this configuration, a transverse electric field promotes lateral circulation, and the resulting mixing intensity increases with the medium’s conductivity [152]. Additional alternating-current phenomena, including AC electroosmosis and induced-charge electroosmosis, further contribute to vortex generation for fluid mixing within microscale domains [148,153]. Designs that use conductive plates or patterned barriers have proven this principle by using electrically induced vortices to achieve almost complete mixing [154,155]. Furthermore, optimization can be achieved by replacing planar plates with conductive plates of irregular or curved geometry, thereby creating an asymmetric flow structure and significantly improving the mixing effect [104,156,157]. Induced charge electrokinetics can also work for non-Newtonian fluids. Pseudoplastic fluids usually mix better than dilatant fluids because they create bigger recirculation zones [154]. A hybrid computational fluid dynamics-artificial neural network approach has even been developed to rapidly predict the electrodynamic flow mixing efficiency of such devices, accelerating the design process [158]. Passive geometrical features such as fixed posts, ridges, or sawtooth patterns can be integrated with electroosmotic flow to promote localized vortices and strengthen interfacial mixing. The efficiency of electrokinetically driven mixing is governed by several interdependent factors, the frequency of the alternating field and the phase delay between voltage cycles [150,159,160]. Adjusting these parameters alters the strength and spatial extent of the induced vortices, shaping how effectively neighboring streams merge. Surface acoustic wave–induced microstreaming is another way to agitate liquids that are trapped without touching them. The acoustic field generates rapid, circulating flow patterns that blend reagents within milliseconds, offering a controllable and contamination-resistant means of achieving uniform microscale mixing [161].

## 6. Electrokinetic Preconcentration and Enrichment

When the concentration of the target analyte is extremely low, the analytical performance tends to drop sharply, which usually limits the performance of analytical and diagnostic systems [162]. When the analyte level is close to the noise floor, some form of enrichment becomes almost unavoidable to secure measurable signals before detection [163]. Electrokinetic techniques provide a practical solution, using field-driven transport to collect or concentrate ionic and even weakly polar species within confined microfluidic regions [164]. This ability to preconcentrate directly on-chip has proved valuable across many areas, from identifying biomarkers associated with disease [165] to tracking low-level pollutants in environmental samples [166].

### 6.1. Ion Concentration Polarization (ICP)

**Figure 6:**
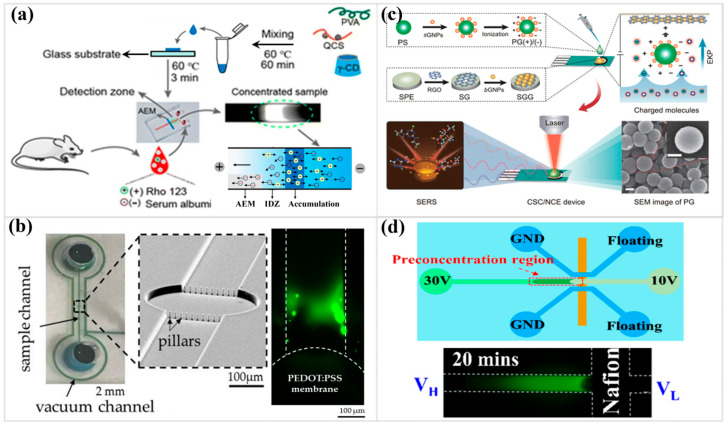
(**a**) Schematics illustration of electrokinetic enrichment principle of a high-performance cation electrokinetic concentrator (Reprinted from Ref. [170] with permission of Royal Society of Chemistry). (**b**) Photographs of the fully assembled concentrator, SEM images of a row of columns, and electrokinetic preconcentration of extracellular vesicles (Reprinted from Ref. [172]). (**c**) Schematic diagram of an electrokinetic pre-concentration-SERS platform based on charge-selective capture/nanoconfined enrichment devices for pesticide analysis (Reprinted from Ref. [191] with permission of ACS). (**d**) Schematic illustration of electrokinetic protein preconcentration operations and fluorescence of FITC-labeled BSA preconcentration (Reprinted from Ref. [188]).

Ion concentration polarization (ICP) [167,168] provides a method for forcibly reshaping the local ionic environment, generating a steep gradient that amplifies minute chemical differences into measurable signals. The depletion–enrichment asymmetry strengthens electrokinetic responses and enables controlled ion transport for preconcentration, desalination, and microscale purification. ICP occurs at the interface between a microchannel and a nanostructured element (such as a nanoporous membrane or a nanochannel) when an electric field is applied. The nanostructure’s surface charge allows it to selectively pass counter-ions while excluding co-ions. This selective ion transport leads to the formation of an ion depletion zone on one side of the nanostructure and an ion enrichment zone on the other [169]. Ion-selective concentration enables charged molecules to be isolated and amplified with unusual precision [170]. The γ-CD/QCS/PVA membrane forms stable ion channels that sustain strong depletion fronts, yielding enrichment factors exceeding 10^5^-fold (Figure 6a). This amplification effect allows cationic substrates, including Rho123, to be detected directly in serum without interference from proteins and requires very little sample.

ICP has evolved into a versatile means of enriching scarce molecular and cellular targets, ranging from proteins and nucleic acids to microbes and mammalian cells [171]. One electrokinetic concentrator built on ICP principles raised extracellular vesicle levels by nearly two orders of magnitude within a short operating window of about half an hour (Figure 6b) [172], a scale of enhancement that often determines whether liquid biopsy signals rise above background fluctuations [173]. Environmental measurements have taken advantage of the same principle, as an ICP module combined with a paper-based colorimetric kit produced enrichment levels close to 156-fold for ionic pollutants—including heavy metals and nitrate—allowing species that once fell below routine detection limits to be brought into view [166]. Furthermore, a method utilizing faradaic ion concentration polarization (fICP) can create a distinct depletion region, thereby enhancing the electric field and drawing charged biomolecules into a confined microbead bed. As nucleic acids accumulate, hybridization reshapes the surface charge landscape, amplifying ion conduction and shifting the current–voltage signature in a logarithmic fashion. Even modest fICP enrichment approaches 10^2^-fold, while established conditions can exceed 10^5^-fold, offering substantial gains for low-voltage, label-free detection [174].

ICP can also be used as an effective means of pre-concentrating and separating charged analytes in paper-based microfluidic devices [175,176,177]. One study [178] elucidates how the acid–base nature of supporting electrolytes governs the electrokinetic stacking performance at ion exchange membranes. This synergistic control of water dissociation, electric field, and pH gradients enables precise enrichment and improved analytical sensitivity in low-cost, portable microfluidic assays. In addition, ICP in the layered paper matrix isolates cfDNA from whole blood and drives it into a sharply enriched zone where electrophoresis and electroosmosis cooperate to elevate local DNA abundance [179]. This concentrated plug then interfaces with CRISPR activation and nanozyme-catalyzed readout, producing pronounced contrast in the lateral flow bands. The combined amplification—ICP enrichment approaching one order of magnitude experimentally and exceeding 10^5^-fold with downstream signal gains—enables detection thresholds down to attomolar levels in both purified samples and whole blood. ICP provides a powerful mechanism for strengthening the chemical contrast required in portable microfluidic paper-based analyses. Simulations and experiments report amplification of more than 100-fold, depending on electrolyte design and flow geometry. These features underscore a unifying strategy for low-cost, field-ready analytical platforms.

### 6.2. Electrokinetic Stacking and Others

Electrokinetic stacking [180,181] is a simple approach to stacking that involves electrokinetically migrating micro- or nanoparticles through moderately confined channels of uniform cross-section. Electrokinetic stacking relies on creating a discontinuity in the electrophoretic velocity of analytes, causing them to slow down and “stack” into a narrow, concentrated band. This result can be achieved by creating a gradient in the electric field, often by changing the cross-sectional area of the channel or by introducing a boundary between buffers of different conductivity. For instance, an electrokinetic stacking chip [182] sharply concentrates a clean pool of fluorophores generated by aptamer-guided recognition and deoxyribonuclease-I recycling. The amplified band gives a stable quantitative readout, which makes it possible to measure α-fetoprotein, carcinoembryonic antigen, and microRNA-21 at clinically relevant trace levels all at once. The dual amplification mechanism offers million-fold effective enrichment, supporting early hepatocellular cancer screening with improved diagnostic confidence. Another application field-enhanced sample stacking combined with micelle-to-cyclodextrin stacking creates a tightly focused enrichment zone that elevates hydrophobic analytes far above their native signal. The dual amplification pathway increases sensitivity by 360 to 1283 times, making it possible to accurately measure sesquiterpenes and alkaloids in complex herbal matrices. The green, solvent-minimized workflow also expands its applicability to a wide range of structurally diverse hydrophobic compounds [183].

Electrokinetic stacking is particularly effective on paper-based microfluidic devices [184,185], where it can be used to significantly improve detection sensitivity. For example, a fast and multiplexed paper-based device with 8 parallel channels has also been designed, allowing for electrokinetic stacking to be completed within 2 min and achieving an enrichment factor of two orders of magnitude. This platform enables online calibration and sample detection on the same chip and has been used for colorimetric detection of food pigments and offline MS/MS detection of amino acids from serum [186]. Similarly, electrodynamic stacking on paper concentrates charged analytes into a distinct region that significantly enhances the surface-enhanced Raman (SERS) signal. Field-amplified gradients, ion-depletion interfaces, and capillary transport jointly compress molecules at the Ag-nanoparticle substrate, lifting SERS intensities by more than an order of magnitude. This confined enrichment enables detection limits as low as 10^−17^ M and reveals ppt-level contaminants directly from aquaculture water, herbal extracts, and food matrices [187]. The rapid, pretreatment-free format broadens practical use in environmental surveillance, food safety, and molecular forensics, while demonstrating how electrokinetic stacking transforms sparsely distributed species into concentrated optical targets with stable, quantifiable signals.

Integrating electrokinetic preconcentration on a microchip steers trace analytes into narrowly confined domains, sharply enhancing their local abundance [177,188,189,190]. The resulting signal becomes stronger and more resilient to matrix interference, allowing rapid, low-volume measurements to reach sensitivities typically reserved for larger, more complex analytical systems [191,192,193]. For instance, ion depletion caused by perm-selective membranes [177,188] creates a sharp barrier where charged particles build up. On the other hand, conductivity gradients in field-amplified stacking [189] squeeze analytes into narrow, high-density plugs. AC-driven thermal and electroosmotic flows (Figure 6c) [191] create a dynamic mechanism that can speed up transport to sensing surfaces, especially in complex matrices where diffusion alone is not enough.

Selective recognition layers—a recurring motif in molecularly imprinted-assisted systems [192,193]—superimpose molecular specificity on top of field-driven migration, ensuring that only structurally relevant analytes are steered into the sensing hotspots. Once these mechanisms migrate into microdevices such as paper-based ICP pads [177], PDMS–Nafion channels (Figure 6d) [188], on-chip concentration polarization-based electrochemical sensing [190], interdigitated microelectrode arrays [191], or graphene-enhanced SERS substrates [192,193], enrichment becomes rapid, voltage-responsive, and spatially defined. Such integration pushes amplification into the 10^2^–10^3^-fold regime, stabilizes optical or capacitive readouts, and enables direct quantification of proteins, polyfluoroalkyl substances, pesticides, alkaloids, and trace pollutants without bulky pretreatment. The resulting architectures reveal how preconcentration techniques can be cleverly combined with microscale materials and electrodynamic control to reshape the sensitivity, selectivity, and usability of modern analytical equipment.

## 7. Emerging Electrokinetic: Electrowetting and Optoelectronic Tweezers

Electrokinetic microfluidics has moved past a purely charge-transport paradigm. In electrowetting systems, droplet motion emerges from electrically defined wetting states, streamlining reaction, transport, and reconfiguration within compact digital layouts [194,195,196]. Optoelectronic tweezers advanced in a different direction, where patterned light is converted into spatially confined electrokinetic forces, enabling soft, parallel manipulation across a wide range of media [197,198,199]. Read together, these approaches point to a microfluidic landscape in which actuation and chemical function are intrinsically intertwined and adaptively expressed [195,197].

### 7.1. Electrowetting-on-Dielectric (EWOD)

Electrowetting-on-dielectric (EWOD) sits at a conceptual boundary between traditional electrokinetic phenomena and digitally controlled microfluidic systems. While droplet motion in EWOD is commonly framed in terms of contact-angle variation, the driving mechanism originates from electrokinetic charge rearrangement at the solid–liquid interface. An applied potential reorganizes the EDL at the dielectric surface, modifying interfacial energy and enabling directed droplet displacement without relying on bulk fluid flow or mechanical actuation [200]. From an electrokinetic perspective, EWOD redirects electric-field action away from bulk ionic conduction and concentrates it within nanometer-scale dielectric interfaces. Forces analogous to electroosmosis and electrophoresis act locally at the solid–liquid boundary, converting spatially uniform fields into stepwise, switchable wetting responses. Such interfacial confinement enables droplet translation, splitting, and fusion to proceed with high positional fidelity. At the same time, convective dispersion and shear-driven fluctuations are largely muted, allowing fluid manipulation that is reagent-efficient, mechanically quiet, and readily scalable with predictable performance [201].

Recent implementations demonstrate that this electrokinetic–electrowetting coupling facilitates intricate chemical processes on planar architectures. EWOD-based digital microfluidics has enabled multi-step bioassays, single-cell isolation, and nucleic-acid amplification by coupling droplet transport with electrokinetically regulated mixing and tightly sequenced reactions [202,203]. Beyond translation, periodic deformation of the liquid–solid interface can generate pressure gradients that sustain pumping without mechanical components, broadening functional scope [204]. Such interfacial dynamics blur conventional boundaries between electrokinetic actuation and flow regulation. In this context, EWOD represents a mode of electrokinetic control centered on reshaping interfaces and fluid states, rather than simply moving droplets. Electric fields are applied to reconfigure interfaces in real time, positioning EWOD at the boundary between electrokinetics and digital microfluidics and enabling integration-ready, digitally addressable chemical operations [196,200].

### 7.2. Optoelectronic Tweezers

Optoelectronic tweezers rely on patterned illumination to shape electric fields, enabling contactless control of objects from the microscale to the millimeter regime. Projected light modulates local conductivity within photoconductive layers, allowing dielectrophoretic force fields to be written, shifted, and erased with high spatial precision and rapid temporal response. This light-addressable control enables parallel transport, positioning, merging, and sorting of droplets, cells, and particles without complex electrode architectures. Recent improvements have made it possible for optoelectronic tweezers to work in oil-immersed and open-chamber formats. The result lowers the amount of optical power needed and makes it easier to use with biochemical assays [205]. Optoelectronic tweezers and their applications are highlighted as a versatile electrokinetic platform where patterned light reshapes electric fields to achieve precise, low-power manipulation. By decoupling optical programmability from mechanical loading, this approach enables concurrent manipulation of cells, particles, and droplets with minimal thermal or photonic stress. The resulting field-programmable control is readily compatible with microfluidic analysis, sensing, and biointerfacing, supporting adaptable chemical and biological microsystems [206].

Connections between optoelectronic tweezers and other field-driven manipulation strategies become clearer when recent optical and electrochemical techniques are examined side by side. Optical tweezers integrated into microfluidic systems have long demonstrated submicron control over particles and single cells, establishing a benchmark for spatial precision. That level of localization, however, is typically accompanied by practical constraints. Operation is often constrained by the need for intense illumination and precise optical alignment, which together narrow the practical parameter space of these systems [207]. Electrochemical and bipolar electrochemical tweezers follow a contrasting physical route. Manipulation arises directly from asymmetric electric polarization, enabling the bending, rotation, or grasping of microobjects without physical contact. Such behaviors point to electric-field-driven charge redistribution as the central origin of microscale motion control, with optical confinement playing no essential role in the actuation process [208]. In analytical microfluidics, optoelectronic tweezers have been coupled with droplet handling, cell positioning, and probing strategies to stabilize targets under flow while preserving compatibility with downstream sensing and measurement modules [209]. Collectively, optoelectronic tweezers exemplify a shift from static, hardware-defined tweezing toward adaptive, field-programmable manipulation, reinforcing their role in emerging intelligent chemical systems where light-defined electrokinetic landscapes coordinate transport, trapping, and analysis.

## 8. Conclusions, Challenges, and Future Outlook

Electrokinetic microfluidics has undergone a gradual but unmistakable transformation over the past decades. Early demonstrations focused almost entirely on electroosmotic flow, electrophoretic motion, and dielectrophoretic trapping. Over time, these mechanisms expanded into a broader engineering language, capable of shaping how microscale assays are built and how signals are drawn from confined liquids. Across a wide range of device embodiments, the imposed electric field no longer serves merely as a means of displacing liquid. Instead, it plays a far more intricate role: reorganizing ionic landscapes, modulating interfacial charge, reshaping velocity fields, and guiding the motion of droplets, particles, and biomolecules with a level of precision that becomes meaningful only at the microscale. As the field continues advancing, these principles have opened pathways to implement high-resolution separations, dynamic mixing, selective preconcentration, and portable diagnostic functions with steadily improving robustness and practicality.

Although electrokinetic microfluidics has advanced considerably, several constraints continue to shape its limits. Among these limitations, consequences linked to intensified electric fields, especially the combined thermal and chemical shifts they induce, frequently become the factors that demand the greatest attention [119]. Joule heating, even when modest, can impose subtle temperature gradients that reshape viscosity, diffusivity, and permittivity across the channel [210]. As the field strength climbs, these gradients intensify and begin influencing the physical stability of polymeric structures and the functional integrity of delicate biological components. When the local temperature rises further, pockets of vapor may nucleate within confined spaces, fragmenting the flow and distorting concentration distributions in ways that are difficult to predict [211].

Electrokinetic systems usually interact with their physical surroundings. Under realistic conditions, strong electric fields and conductive media inevitably trigger localized Joule heating, subtly reshaping flow profiles, weakening interfacial stability, and narrowing the range of biologically tolerable operation [212]. As operating times extend, material-related issues surface: electrodes age, side reactions accumulate, and DC-driven architectures become particularly susceptible to electrochemical drift. The problem is further complicated by the samples themselves. Ionic strength, viscosity, and protein abundance—hallmarks of biological matrices—continuously redefine interfacial charge states, dampen transport efficiency, and promote fouling at active surfaces. These effects are not merely incremental. When devices are scaled or arranged in parallel, maintaining uniform fields, dissipating heat, and preserving electrode integrity turn into dominant design constraints rather than secondary considerations.

A second horizon of difficulty arises from the fundamental mismatch between idealized working media and real-world samples. Many electrokinetic models assume Newtonian liquids, uniform surface charge, and well-behaved ionic solutes. In contrast, most analytical applications involve non-Newtonian, viscoelastic, protein-rich, or particulate-laden matrices. Blood, serum, cell culture media, environmental extracts, and intricate food or herbal components all display rheological characteristics that substantially disrupt electrokinetic transport [133]. Surfactants and amphiphilic additives—often included for biocompatibility or stability—can unpredictably alter zeta potential, suppress mobilities, and modify double-layer structure [43]. These complex factors affect the movement of droplets, their concentration, how they mix in small areas, and the stability of induced charge electroosmotic vortices [130,131,134]. A deeper theoretical understanding, supported by constitutive models that fully capture viscoelastic relaxation, shear-thinning behavior, nonlinear electrohydrodynamics, and interfacial ion exchange, will be essential for translating electrokinetic technologies into broader analytical contexts [210].

Translating microfluidic concepts from the bench to practical use presents persistent challenges. The chip may be small, often deceptively so, but the equipment surrounding it rarely matches that scale. High-voltage generators, bench microscopes, multichannel pumps, and research-grade optical modules accumulate into a footprint far larger than the device they support. Such auxiliary hardware adds cost, demands controlled environments, and ultimately restricts the technology’s mobility beyond specialized laboratories. Encouraging signs of progress can already be seen: triboelectric nanogenerators [213,214,215] capable of powering field-driven transport, handheld electrophoresis analyzers [63], and smartphone-assisted readout schemes demonstrate that portable [166,216], self-powered, or low-powered [217,218] architectures are feasible. Fabrication techniques have also undergone diversification. 3D-printed microchannel scaffolds, paper-based layered systems, and modular “plug-and-run” blocks have all expanded the range of accessible device platforms, including printed circuit board designs [219]. Future advances will likely depend on unifying these elements into coherent systems where sample handling, preconcentration, separation, detection, and data interpretation occur seamlessly within a single integrated layout [2,64].

The growing interplay among computation, artificial intelligence, and experimental electrokinetics has created a landscape that feels markedly different from even a few years ago [158]. Numerical modeling, once relied upon mainly for mapping field distributions or tuning channel geometries, now intersects with learning algorithms that compress lengthy optimization routines into far shorter cycles [220]. In several cases, neural networks have already demonstrated an ability to estimate mixing performance or anticipate the onset of electrohydrodynamic instability at a fraction of the usual computational burden [158]. As device layouts continue to accumulate complexity, such data-driven tools may evolve into quiet but decisive influences—shaping parameter choices, steering geometric adjustments, and supporting more adaptive forms of control [221].

Materials innovation is becoming a central force in reshaping electrokinetic platforms. The field is gradually moving beyond familiar glass and PDMS toward hybrid constructs that make use of functional polymers, nanocomposite films, ion-selective membranes, high-stability electrode coatings, and surfaces engineered for controlled chemical reactivity [43,93]. Such materials open space for subtler tuning of zeta potential, stronger charge retention under demanding electric fields, and more selective ionic transport—features that collectively extend electrokinetic behavior far beyond the limits imposed by silica-based interfaces [45]. At the same time, nanostructured surfaces with adjustable slip length, tailored hydrophobicity, or confined transport pathways are revealing new possibilities for reinforcing electroosmotic flow, shaping induced-charge polarization, and elevating preconcentration performance.

From a physical standpoint, EOF, DEP, electrohydrodynamic instability, and electrokinetic enrichment are not independent operating regimes but different expressions of the same underlying electrokinetic framework. At low field strengths and near-uniform charge distributions, transport follows linear electroosmotic and electrophoretic responses. As spatial charge imbalance, electric-field gradients, and ion depletion intensify, nonlinear flow patterns and localized enrichment emerge naturally. Across these regimes, system behavior is dictated by the same physical controls—namely, interfacial charge arrangement, spatial variations in the electric field, and coupled fluid-ion dynamics imposed by confinement.

To distinguish between validation studies and more practically applicable approaches to electrokinetic microfluidic devices, their approximate Technology Readiness Levels (TRLs) are elucidated. Droplet electrodynamics, including the transport and breakup of Janus droplets, is defined as mechanism-driven proof-of-concept work (TRLs 2–4), elucidating interfacial electrohydraulic dynamics but remaining sensitive to chemical properties and electric field homogeneity [37,49,50,51,52,53]. In contrast, electrokinetic injection and microcrystal electrophoresis exhibit higher readiness (TRLs 4–7) and have examples of integrated separation and portable analyzers suitable for wafer lab deployments [63,64]. Electrokinetic preconcentration strategies, particularly ICP and stacking on paper-based or hybrid wafers, are considered most relevant to point-of-care testing (POC) (TRLs 5–9) and have been validated using whole blood, serum, and field-compatible readout formats [179,186,188,190,191,212,213,214]

In summary, electrokinetic control has shifted from a transport mechanism into a means of extracting subtle chemical and biological signals from tiny volumes. Electric fields now change the motion of droplets and ionic gradients with such accuracy that they can replace mechanical assemblies and speed up the time it takes to analyze data. Field-driven enrichment on lightweight substrates, on the other hand, finds contaminants that traditional tests have been hiding for a long time. Electrohydrodynamic forcing has quietly reshaped how reactions proceed in water, guiding nanoparticle arrangements without harsh reagents. Recognizing these mechanisms as interconnected responses enables electrokinetic microfluidic systems to be designed more rationally, where transport, mixing, and enrichment can be deliberately coupled rather than independently optimized. Advances in numerical modeling, machine-learning design, interface engineering, and data-driven device tuning are making operations less complicated. As electrokinetic responses become more predictable in soft and heterogeneous matrices, the technology is moving toward compact, field-ready platforms capable of real-time sensing, purification, and microscale chemical transformation.

## Figures and Tables

**Table 1:** Mathematical models of EOF, EP, and DEP in electrokinetic microfluidics.

Mechanism	Governing Basis	Representative Equation	Key Dependent Parameters	Scaling Nature	Typical Use/Dominant Regime
EOF	Stokes flow + electrical double layer charge	$u_{EOF}=-\frac{\varepsilon \zeta }{\mu }E$	permittivity *ε*, viscosity *μ*, wall zeta potential ζ	Linear scaling with *E*	Pumping/Bulk fluid transport
EP	Charged particle drift balancing electrostatic force and viscous drag	$u_{EP}=$ *μ_EP_ E* $=\frac{\varepsilon \zeta_{P}}{\mu }E$	particle zeta potential ζ_*p*_, medium *ε*, viscosity *μ*	Linear scaling with *E*	Molecular/Particle separation
DEP	Field-induced dipole polarization in non-uniform AC (or DC) fields	*F_DEP_* = 2*πr*^3^*ε_m_ Re* [K(*ω*)] ∇∣*E*∣^2^	particle radius *r*, permittivity *ε_m_*, Clausius–Mossotti factor K(*ω*), real part of K(*ω*) *Re* [K(*ω*)]	Nonlinear scaling with *E*	Label-free manipulation/Trapping and sorting

## Data Availability

The data presented in this study are available upon request from the corresponding author.
